# Factors Associated with Leptospirosis in Domestic Cattle in Salakphra Wildlife Sanctuary, Thailand

**DOI:** 10.3390/ijerph16061042

**Published:** 2019-03-22

**Authors:** Nantawan Yatbantoong, Rattanawat Chaiyarat

**Affiliations:** 1 Department of Large Animal and Wildlife Clinical Science, Faculty of Veterinary Medicine, Kasetsart University, Kampheangsean Campus, Kampheangsean 73140, Thailand; nantawan@yahoo.com; 2 Wildlife and Plant Research Center, Faculty of Environment and Resource Studies, Mahidol University, Nakhonpathom 73170, Thailand

**Keywords:** domestic cattle, leptospira, livestock production, protected area

## Abstract

Leptospirosis found in cattle (*Bos taurus indicus*) has potentially increased in economic impact. The objective was to investigate the factors associated with leptospirosis in cattle in the protected area. We investigated the seroprevalence of leptospirosis in cattle in Salakphra Wildlife Sanctuary, Thailand. Serum was collected to investigate the seroprevalence by agglutination test and their associated factors. From a total of 513 samples, antibodies against Leptospira were detected in 92.2% of samples. Within a total of 42 herds, the serovar with the highest prevalence was *L interrogans* serovar Tarassovi (92.9%). Most leptospirosis was found in medium-sized herds with the highest concentrations in cattle farms close to cities (52.4%, *p* < 0.05). Seroprevalence was associated with herd size, raising pattern in the dry and wet seasons, grazing distance, number of years that cattle were kept in the farm, the introduction of new cattle into the farm, and keeping some pets in the farm. The results of the study suggest that keeping cattle in larger herds, raising pattern and distance, keeping period, and introducing new cattle and having pets impart potential risk of increasing leptospirosis exposure. These results indicate that cattle are important hosts of Leptospira in Thailand and may act as sentinels of Leptospira infection for wildlife and people in the protected areas.

## 1. Introduction

Leptospirosis affects humans worldwide (Haake and Levett, 2015). Occupations involving exposure to domestic cattle (*Bos taurus indicus*) (e.g., farm workers, abattoir workers, and veterinarians) is a risk factor for human leptospirosis [1]. Thailand has a high incidence of leptospirosis relative to other tropical Southeast Asian countries. Most cases of leptospirosis are found during the rainy season in northeast Thailand [2,3]. Previous surveys have found a correlation between circulating anti-leptospira antibodies and occupational exposure, travel to endemic areas with recreational activities, and small dam construction by park rangers [4,5]. Leptospirosis is a well-recognized cause of abortions, stillbirths, and the births of weak offspring in domestic cattle [6]. In tropical countries, bovine leptospirosis is endemic, placing a hard burden on agribusiness and related to economic hazards [7,8]. Infected animals may excrete Leptospira intermittently or regularly for months, years, or their entire lifetime [9]. The infection in domestic cattle has been associated with abortion in late pregnancy, stillbirth and low fertility [10]. The severity of the disease can vary from unapparent to fatal depending on the host involved and the seroprevalence [11]. Leptospira can survive outside the body if environmental conditions are favorable [10]. Infectious urine and body fluids constitute two of the major sources of infection [11].

The number of domestic cattle in several protected areas of the world has been increasing [12]. Grazing in these areas causes food and habitat competition and possibly introduces disease to wildlife [13]. The sharing of habitat between domestic cattle and other animals has been found to be one of the main causes of leptospirosis transmission [14,15,16].

Salakphra Wildlife Sanctuary is located in the Western Forest Complex (WEFCOM) (>11,700 km^2^) in Thailand and is surrounded by farmland. Indeed, 50% of this protected area is invaded by domestic animals. Chaiyarat and Srikosamatara [17] found that the population of domestic cattle in Salakphra Wildlife Sanctuary was greater than 16,000 individuals based on interviews. The number of domestic cattle is still increasing dramatically (P. Prempree, asst. head of Salakphra Wildlife Sanctuary, personal comm.). The aim of this study was to investigate if cattle reared in a protected wildlife area can act as a sentinel of Leptospira infection for wildlife and people.

## 2. Materials and Methods

### 2.1. Study Area

**Figure 1:**
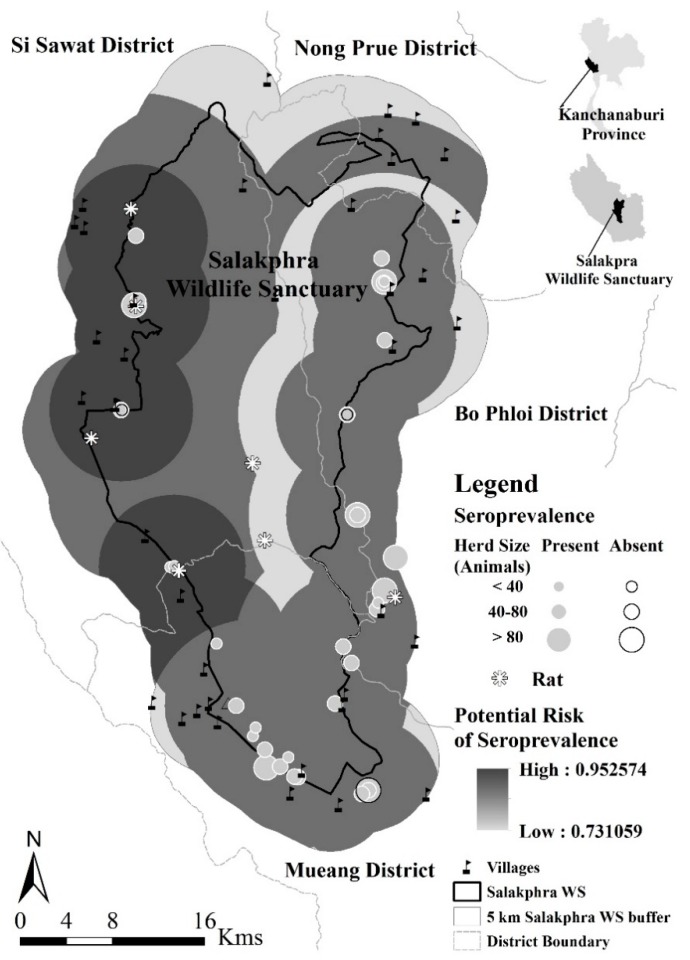
The location of cattle and the seroprevalence along the Salakphra Wildlife Sanctuary.

Salakphra Wildlife Sanctuary is located in Kanchanaburi province (14.9º–14.41º N–99.10º–99.25º E; total area of 858.6 km^2^) (Figure 1). The area can separated into wet (May–October) and dry (November–April) seasons. The area is composed of mixed deciduous, dry dipterocarp, and dry evergreen forest; in which Asian elephant (*Elephas maximus*), gaur (*Bos gaurus*), and sambar deer (*Rusa unicolor*) can be found [18].

### 2.2. Study Population

Animal treatment protocols were approved by the Ethics Committee of Mahidol University, Thailand in 2017. The study was conducted on cattle herds totaling more than twelve thousand animals based on estimates by park rangers. The sample size for this study was estimated by WinEpiscope 2.0 (Zaragoza, Spain) at 10% of the total individuals in each cattle herd with 5% allowable error. At least 254 of the animals should be included in the study to achieve a 95% confidence interval. In this study, a total of 513 domestic cattle were randomly selected.

#### 2.2.1. General Information on Domestic Cattle Keeping

All 42 of the selected herds located in the 5 km boundary of Salakphra Wildlife Sanctuary were visited in this research, resulting in a 100% response rate for participation in the study. The average herd size was 62±32 animals. The primary purpose of raising domestic cattle was for sale to the local slaughterhouses (95.2%). All herds had feeding supplements such as hay and mineral blocks. For healthcare management, 97.6% of herds were vaccinated with foot-and-mouth disease vaccine and 85.7% used ivermectin for deworming routinely. Sixty-two percent of the herds had a sign of sickness such as lameness, wounds, and hoof problems.

#### 2.2.2. Sample Collection and Seroprevalence Examination

Blood samples of domestic cattle were collected by jugular venipuncture between April 2009 and November 2010 (Table 1). All serum samples were examined for *Leptospira interrogans* antibodies by means of the Microscopic Agglutination Test (MAT) as previously described [19]. Twenty different serovars of *Leptospira interrogans* were tested: *L. interrogans* serovar Tarassovi, Ranarum, Hebdomadis, Ballum, Bratislava, Sejroe, Autumnalis, Pyrogenes, Bataviae, Sarmin, Canicola, Djasiman, Icterohaemorrhagica, Pomona, Cynopteri, Mini, Javanica, Louisiana, Shermani, and Patoc I (listed in Table 2). *L interrogans* serovar Tarassovi is commonly found in the area [20]. Individual sera were considered positive if agglutination was present at dilutions of 1:50 or more [21], and a herd was considered seropositive when at least one animal tested as seropositive in a certified laboratory at the Faculty of Veterinary Medicine, Kasetsart University, Kampheangsean Campus.

Rabbit hyperimmune sera were raised individually against the 20 serovars by weekly intravenous injection for 4–6 weeks [22]. The MAT was used to detect antibody titers to live leptospiral cultures on microtiter plates [23]. Antisera with high agglutinating titers (greater than 12,800) were collected and used in conjugation. Briefly, serum globulins were fractionated by ammonium sulfate precipitation and labeled with fluorescein isothiocyanate (FITC) dye [24]. Unbound proteins and excess free dye were removed by Sephadex gel filtration and tissue absorption. The resulting fluorescein-labeled antibody conjugates were predetermined and optimized with smears of reference cultures before use. The reactivity and specificity of the test was determined at a final dilution of conjugate that gave a strong fluorescence with target antigens of the homologous leptospires and no staining with the heterologous or unrelated strains particular to the different serogroups. To identify leptospire infection, kidney tissues were cryosectioned at 4–5 μm thick and fixed in cold acetone for 5 min before drying at room temperature. Sections were stained with appropriate dilutions of individual fluorescent conjugates for 30 min. After 15 min of washing off the excess conjugate, the sections were mounted and examined under a fluorescent microscope (Fluophot, Japan) equipped with a filter set for FITC. A positive finding on DFA of kidney revealed a yellowish-green fluorescence to the spiral leptospirae, which was distinguishable from the dark background of the surrounding tissues. The isolates recovered were serotyped by MAT at the local laboratory and compared with the results of the cross agglutinin absorption test (CAAT) of the corresponding isolates carried out at the WHO/FAO/OIE Collaborating Center for Reference and Research on Leptospirosis, Australia.

The diversity index (*H’*), rarefaction, and similarity index (Kulczynski comparison) were calculated by using BioDiversity Professional Version 2 [25].

### 2.3. Statistical Analysis

ArcGIS version 10 (ESRI, USA) was used for mapping the relationships between environmental factors and seroprevalence in domestic cattle (Table 3). Information about the raising distances from farms in Salakphra Wildlife Sanctuary, the number of years that the domestic cattle were kept in the farms, the introduction of new animals into the herds, sharing the same routes with other herds, sharing the feeding grounds with other herds, and abortion histories were collected from interviews with the herd owners.

The Shannon–Wiener diversity index [26], used to examine seroprevalence and indicate the level of seroprevalence diversity in cattle and red spiny rat (*Maxomys surifer*), is as follows

(1) $$H^{\prime }=\sum_{i=1}^{S}p_{i}\log p_{i}$$

where *H′* = Diversity index of Shanon-Weiner. *p_i_* = The proportions of the many capabilities of the seroprevalence *i* compared to all of the seroprevalence (*N*) = *n_i_*/*N* when *i* = 1, 2, 3,…, S. *S* = number of seroprevalence in cattle

A chi-squared test was employed to test differences between leptospirosis and the associated environmental factors. Differences were determined to be significant based on the *p* < 0.05. Statistical analyses were performed using SPSS. The relationships between the herd variables and the seroprevalence were analyzed via the correlative coherence analysis (CCA) with PC-ORD 5.10 [27].

## 3. Results

### 3.1. Serological Prevalence to Leptospirosis

Of the total 513 serum samples analyzed, 473 samples (92.2%) had antibodies against at least one of the 20 Leptospira serovars, which were used at a titer equal to or above 1:50. Overall, a total of 42 herds, the serovar with the highest herd seroprevalence was *L interrogans* serovar Tarassovi (92.9%). The highest titer observed was 1:800 (Table 2). The potential high risk of transmission, detected through red spiny rat, was found mainly in the villages and Wildlife Sanctuary Guard Stations in Si Sawat district (Figure 1).

Only 7.1% of positive herds had a single serovar and the remaining 92.9% had more than two serovars (Table 2). Approximately 52.4% were kept in a medium-sized herd. The large herd sizes were located mostly in Mueang district, which had the highest concentration of domestic cattle farms close to the city. While Si Sawat and Bo Phloi districts were lower in numbers of farms and domestic herd sizes (*χ*^2^ = 44.56, df = 6, *p* < 0.001), the presence of leptospirosis was not different among districts (Figure 1).

### 3.2. Potential Disease Transmission

The herd sizes were different among the households. Most animals were kept in medium size herds (40–80 individuals; 52.4%) (*χ*^2^ = 7, df = 2, *p* = 0.03). There was no difference in raising patterns of domestic cattle between dry and wet seasons. In both seasons, a few herds were kept in the village and most of them were moved to the forest for free grazing. The raising distances from farms in Salakphra Wildlife Sanctuary were different. Most herds were raised in distances of more than 5 km (*χ*^2^ = 18.8, df = 3, *p* < 0.001). The number of years that the domestic cattle were kept in the farm was different. Most of them were kept longer than 10 years (*χ*^2^ = 32.5, df = 4, *p* < 0.001). Introduction of new animals into the herd (*χ*^2^ = 9.5, df = 1, *p* = 0.002) and pets on the farms (*χ*^2^ = 18.7, df = 1, *p* < 0.001) were different. However, the category raising the same route with other herds, sharing the feeding ground with other herds, and abortion histories were not different (Table 1).

**Figure 2:**
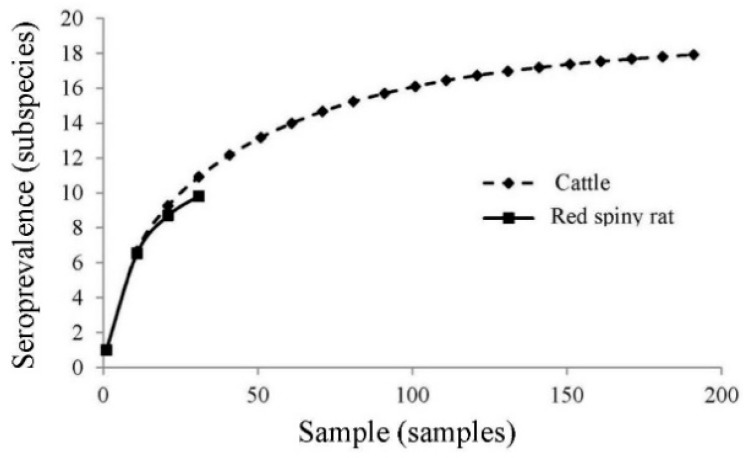
Seroprevalence accumulation curves of cattle and red spiny rat in Salakphra Wildlife Sanctuary.

The diversity of seroprevalence in cattle (*H*′ = 1.027, n = 18 serovars) was higher than red spiny rat (*H*′ = 0.905, n = 10 serovars) with *χ*^2^ = 173.4 (df = 2, *p* < 0.001). The similarity between cattle and red spiny rat were 62.2% (Table 2), the rarefaction is shown in Figure 2.

The relationship between the herd seroprevalence (Table 2) and the potential risk factors of disease transmission was related to raising distance (Table 1). The herds that grazed deeper in the forest had a higher potential infection rate than those that were kept close to the village (*χ*^2^ = 8.9, df = 3, *p* = 0.03).

**Figure 3:**
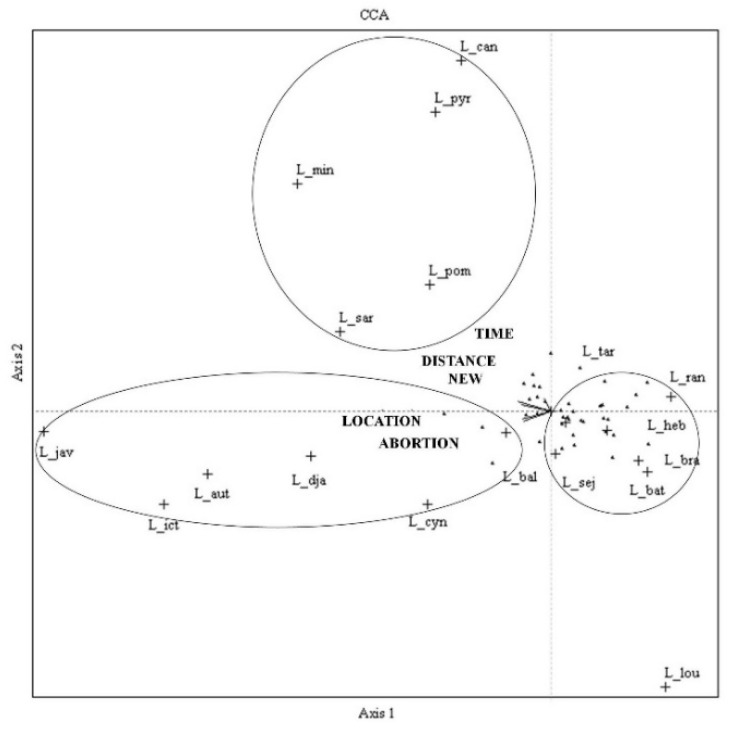
Relationships of 18 seroprevalence and herd variables revealed of domesticated cattle by Correlative Coherence Analysis (CCA) in the Salakphra Wildlife Sanctuary, TIME = Number of years that livestock were kept in the farm (years), DISTANCE = Raising distance (km/day), NEW = Introduced of new animal into herd, LOCATION = Raising in the same route with other herds, ABORTION = Abortion history, L_aut = Autumnalis, L_bal = Ballum, L_bat = Bataviae, L_bra = Bratislava, L_can = Canicola, L_cyn = Cynopteri, L_dja = Djasiman, L_heb = Hebdomadis, L_ict = Icterohaemorrhagica, L_jav = Javanica, L_lou = Louisiana, L_min = Mini, L_pom = Pomona, L_pyr = Pyrogenes, L_ran = Ranarum, L_sar = Sarmin, L_sej = Sejroe.

Serovars Canicola, Mini, Pomona, Pyrogenes, and Sarmin were correlated with the number of years that domestic cattle were kept in the herds, the distance of raising from the households, and the location of herds. While serovars Autumnalis, Ballum, Cynopteri, Djasiman, Icterohaemorrhagica, and Javanica were associated with the introduction of new animals into the herds and the abortion history of the herds (Table 4 and Figure 3).

## 4. Discussion

The seroprevalence found in this study was higher than a recent survey of cattle (17%) of nearby protected areas in Kanchanaburi province [20] as well as a survey of cattle (9.9%) conducted in Thailand by Suwancharoen et al. [28]. The high prevalence suggests that leptospirosis may be endemic in the study area.

The seroprevalence in this study did not match previous studies in domestic cattle in Thailand by Laoluekiat and Jongpipatvanitch [20], who found that antisera to serovars Shermani and Ranarum (79%) were most prevalent in cattle. In this study, Serovars Tarassovi and Ranarum were most prevalent in cattle (>80%) in the Salakphra Wildlife Sanctuary. They were a major caused of sickness in daily cow in the central part of Thailand [29]. Mammals can be found with one or more Leptospira species and the prevalence of various species differs considerably depending on the country, district, or season. Furthermore, the number of serovars found may be different among age classes. Actually, seroprevalence in young domestic cattle has been reported to be lower than in older domestic cattle [30]. Maintenance of Leptospira in these populations was due to their continued exposure to animal reservoirs or transmission within animal herds [31]. Another reason for a high seropositive number of animals with one or more Leptospira reactivities in this study may be due to animals carrying mixed species exposures or a cross-reactivity between different Leptospira serogroups [10]. It is important to take into account the results with multiple serovars and note the frequency of cross reactions to early serology. There is a high risk of nonspecificity of MAT at the beginning of infection. In this study, seroprevalence in cattle was higher than red spiny rat and the similarity between cattle and red spiny rat were 62.2%. While, a previous study found the highest leptospirosis seroprevalence among the animals tested was in rodents [32], which is completely different from the seroprevalence found in this study. Indeed, there are reports of seroprevalence that do not correspond to rodents [14] that may be a maintenance host or carrying pathogens for *Leptospira* spp. The real prevalence (by direct identification of the agent, PCR, or isolation) was probably the opposite. It could be suggested that the diseases were not transmitted only by rodents and domestic cattle but could be an incidental infection caused by strains carried by other domestic or free-living animals [33]. However, the high seroprevalence in domestic cattle in this study is similar to studies of domestic Asian elephants in Thailand [34] and India [35]. Leptospira pathogens have a high potential to be transmitted from domestic cattle to wild Asian elephants as well as wildlife to domestic cattle in Salakphra Wildlife Sanctuary due to the high percentage of seroprevalence in animals along the buffer zone of the sanctuary. It is probable that elephants may help transmit leptospirosis within domestic cattle [34].

Management factors have potentially influenced the transmission between domestic cattle and red spiny rats. Cattle raised in herds for over ten years faced a higher risk-the longer the infected animals remained in contact with the remainder of the herd, the greater the number of seropositive animals [36]. For a medium-sized herd, high stock density or a confined population might confer high risk of infection within the herd [10]. Free grazing distances in the forest of more than 5 km from a farm increase the potential of transmission of pathogenic serovars such as Shermani and Patoc I from rodents in the inner forest areas as well as pets such as dogs [37], but it does not demonstrate the role of dog as a carrier. Dog urine is acidic and Leptospira is usually rapidly inactivated in their urine. Dogs are known to be chronic carriers just for serovar Canicola [38]. Whereas, a host factor such as abortion history, which is a clinical manifestation of seroprevalence, was not a potential risk. The seroprevalence found in this study indicates the Leptospira were adapted to animals as their preferred host—adapted seroprevalence rarely results in acute and severe disease [10].

Finally, our study supports the idea that keeping domestic cattle within an area with a high risk of contact with other animals has a potential risk of leptospirosis transmission among domestic cattle [39]. The potential infection between domestic cattle and red spiny rats are increasing due to various grazing distances into the forest. Leptospirosis is a reemerging infectious disease, and one of the causes of this appearance is the climatological conditions. In tropical areas with long rainy seasons, the cases increase [16]. We suggest that red spiny rats within the grazing range in the forest should be surveyed for the potential risks of transmission.

## 5. Conclusions

Most domesticated cattle in the Salakphra Wildlife Sanctuary that were kept in medium-sized herds had higher seroprevalence than other herd sizes. *L. tarassovi*, *L. ranarum*, and *L. hebdomadis* were most commonly found in the area. These species are also found in a domestic Asian elephant camp nearby the Salakphra Wildlife Sanctuary. Other environmental factors that were associated with leptospirosis in the Salakphra Wildlife Sanctuary were domestic cattle raising patterns, raising distance, keeping periods, the introduction of new animals into the herds, other pets kept on the farms, and distance from villages. We suggested that domestic animals should be fenced away from sharing habitat with other wildlife to reduce the potential transmission of Leptospira in the area and elsewhere.

## Figures and Tables

**Table 1:** Herd size, sample size, location and seroprevalence of *Leptospira interrogans* found in domestic cattle in Salakphra Wildlife Sanctuary.

Herd ID	Herd Size (Individuals)	Sample Size (Individuals (%))	Location	Seroprevalence of *Leptospira interrogans* Serovars																	
				aut	bal	bat	bra	can	cyn	dja	heb	ict	jav	lou	min	pom	pyr	ran	sar	sej	tar
A	70	7 (10)	1	0	0	0	0	0	0	0	4	0	0	0	0	0	0	7	0	0	7
B	60	7 (11.7)	1	0	0	0	1	0	0	0	3	0	0	0	0	0	0	7	0	0	7
C	30	5 (16.7)	1	0	0	0	0	0	0	0	0	0	0	0	0	0	0	5	0	0	5
D	70	5 (7.1)	1	0	0	0	3	0	0	0	2	0	0	0	0	0	0	5	0	0	5
E	14	9 (64.3)	1	0	9	0	3	0	1	1	3	0	0	0	0	0	0	8	0	5	9
F	7	7 (100)	1	0	6	1	2	0	0	0	2	0	0	0	0	1	0	6	0	5	6
G	9	2 (22.2)	1	0	0	0	1	0	0	0	1	0	0	0	0	0	0	2	0	0	2
H	42	2 (4.8)	2	0	0	0	2	0	0	0	0	0	0	0	0	0	0	2	0	0	2
I	44	9 (20.5)	2	0	0	0	5	0	0	0	5	0	0	0	0	0	0	9	0	0	9
J	70	4 (5.7)	2	0	0	0	0	0	0	0	0	0	0	0	0	0	0	0	0	0	0
K	37	3 (8.1)	2	0	0	0	1	0	0	0	1	0	0	0	0	0	0	3	0	0	3
L	80	1 (1.3)	2	0	0	0	0	0	0	0	0	0	0	0	0	0	0	1	0	0	1
M	120	2 (1.7)	2	0	0	0	0	0	0	0	0	0	0	0	0	0	0	2	0	0	2
N	60	4 (6.7)	2	0	0	0	0	0	0	0	1	0	0	0	0	0	0	4	0	0	4
O	24	3 (12.5)	2	0	0	0	0	0	0	0	1	0	0	0	0	0	0	3	0	0	2
P	27	3 (11.1)	1	0	0	0	0	0	0	0	0	0	0	0	0	0	0	3	0	0	3
Q	42	4 (9.5)	1	0	0	0	1	0	0	0	0	0	0	0	0	0	0	4	0	0	4
R	59	6 (10.2)	1	0	0	0	0	0	0	0	1	0	0	0	0	0	0	5	0	0	4
S	60	6 (10)	3	0	0	0	1	0	0	0	1	0	0	0	0	0	0	6	0	0	6
T	60	2 (3.3)	3	0	5	0	0	0	0	0	1	0	0	0	0	0	0	3	-	-	3
U	31	7 (22.6)	3	0	5	0	0	0	0	0	1	0	0	0	0	0	1	5	1	2	7
W	70	7 (10)	3	0	0	0	0	0	0	0	0	0	0	0	0	0	0	0	0	0	0
X	110	4 (3.6)	3	0	0	0	0	0	0	0	1	0	0	0	0	0	0	0	0	0	0
Y	50	6 (12)	3	0	2	0	0	0	0	0	1	0	0	0	0	0	0	1	0	1	2
Z	143	17 (11.9)	3	0	14	1	1	0	0	0	2	0	0	0	0	0	0	11	0	2	13
AA	34	5 (14.7)	3	0	2	0	0	0	0	0	0	0	0	0	0	0	0	4	0	0	4
BB	65	8 (12.3)	3	0	6	0	0	0	0	0	3	0	0	0	0	0	0	6	0	0	6
CC	100	15 (15)	3	0	9	0	2	0	0	0	5	0	0	0	0	0	1	14	0	0	15
DD	70	7 (10)	1	0	1	0	0	0	0	0	0	0	0	0	0	0	0	6	0	0	6
EE	130	1 (0.8)	1	0	0	0	0	0	0	0	0	0	0	0	0	0	0	0	0	0	0
FF	100	10 (10)	1	0	4	0	1	0	0	0	2	0	0	0	0	0	0	9	0	0	1
GG	100	4 (4)	1	0	2	0	0	0	0	0	0	0	0	0	0	0	0	2	0	0	3
HH	40	5 (12.5)	1	0	2	0	0	0	0	0	0	0	0	0	0	0	0	5	0	2	5
II	57	8 (14)	1	0	2	0	0	0	0	0	1	0	0	0	0	0	0	1	0	2	4
JJ	107	10 (9.3)	1	0	4	0	1	0	0	0	4	0	0	0	0	0	0	4	0	5	9
KK	33	10 (30.3)	1	0	10	0	0	0	0	0	1	0	0	1	0	0	0	10	0	9	9
MM	60	20 (33.3)	1	2	12	0	0	9	0	0	2	0	0	0	1	1	13	13	2	3	13
TT	40	13 (32.5)	3	5	5	0	0	1	0	1	1	0	0	0	0	1	0	1	2	0	7
UU	47	10 (21.3)	3	4	4	0	0	0	0	0	1	0	0	0	0	0	0	0	0	0	7
VV	60	12 (20)	3	12	12	0	3	1	1	2	3	0	1	0	1	0	3	0	0	4	12
WW	105	18 (17.1)	3	13	13	0	1	0	0	0	2	2	0	0	0	0	1	2	2	4	18
XX	2,537	225 (8.9)	1	36	129	2	29	11	2	4	56	2	1	1	2	3	19	179	7	44	225
Seroprevalence (individuals (%))				72 (14)	258 (50.3)	4 (0.8)	58 (11.3)	22 (4.3)	4 (0.8)	8 (1.6)	112 (21.8)	4 (0.8)	2 (0.4)	2 (0.4)	4 (0.8)	6 (1.2)	38 (7.4)	358 (69.8)	14 (2.7)	88 (17.2)	450 (87.7)

Location 1 = Muang district, 2 = Sri Sawat district, 3 = Bo Phloi district, aut = Autumnalis, bal = Ballum, bat = Bataviae, bra = Bratislava, can = Canicola, cyn = Cynopteri, dja = Djasiman, heb = Hebdomadis, ict = Icterohaemorrhagica, jav = Javanica, lou = Louisiana, min = Mini, pom = Pomona, pyr = Pyrogenes, ran = Ranarum, sar = Sarmin, sej = Sejroe, Tar = Tarassovi.

**Table 2:** Seroprevalence and the Shannon–Wiener diversity index of twenty *Leptospira interrogans* serovars found in domestic cattle herds (n = 42 herds) and red spiny rats (n = 70 sites) in Salakphra Wildlife Sanctuary.

Serovars	Cattle Herd (% Prevalence)	Red Spiny Rat (% Prevalence)	Titer Range	*χ* ^2^	df	*p*-value
*Leptospira interrogans*						
Tarassovi	39 (92.9)	4 (30.8)	50–800	28.5	1	<0.001
Ranarum	37 (88.1)	4 (30.8)	50–400	26.6	1	<0.001
Hebdomadis	30 (71.4)	-	5–800	30	1	N/A
Ballum	22 (52.4)	-	50–400	22	1	N/A
Bratislava	17 (40.5)	-	50–400	17	1	N/A
Sejroe	12 (28.6)	1 (7.7)	50–400	9.3	1	0.002
Autumnalis	6 (14.3)	-	50–100	6	1	N/A
Pyrogenes	6 (14.3)	-	50–400	2	1	0.153
Bataviae	5 (11.9)	2 (15.4)	50–100	5	1	N/A
Sarmin	5 (11.9)	1 (7.7)	50–100	2.7	1	0.098
Canicola	4 (9.5)	4 (30.8)	50–100	0	1	N/A
Djasiman	4 (9.5)	-	50	4	1	N/A
Icterohaemorrhagica	3 (7.1)	-	50–100	3	1	N/A
Pomona	3 (7.1)	3 (23.1)	50	0	1	N/A
Cynopteri	2 (4.8)	1 (7.7)	5–100	0.3	1	0.571
Mini	2 (4.8)	-	50	2	1	N/A
Javanica	1 (2.4)	-	100	1	1	N/A
Louisiana	1 (2.4)	-	50	1	1	N/A
Shermani	-	9 (69.2)	50	9	1	N/A
Patoc I	-	4 (30.8)	50	4	1	N/A
*H*′	1.027	0.905		173.4	40	<0.001

Leptospires used for rabbit immunization.

**Table 3:** The relationships between environmental factors and seroprevalence in domestic cattle in Salakphra Wildlife Sanctuary.

Environmental Factor	Level	Seroprevalence *n* (%)	*χ* ^2^	df	*p*-Value ^a^
Herd size (individuals)	1 = small (<40) 2 = medium (40–80) 3 = large (>80)	11 (26.2) 22 (52.4) 9 (21.4)	7	2	0.03 *
Raising patterns - Dry season - Wet season	1 = free grazing 2 = stationed 1 = free grazing 2 = stationed	40 (95.2) 2 (4.8) 34 (80.9) 8 (19.1)	42.9 67	1 1	<0.001 *** <0.001 ***
Raising distance (km/day)	1 = <1 2 = 2–3 3 = 4–5 4 = >5	3 (7.1) 9 (21.4) 8 (19.1) 22 (52.4)	18.8	3	<0.001 ***
Water resource	1 = natural source 2 = artificial ponds	23 (54.8) 19 (45.2)	1.5	1	0.22 ^ns^
Number of years that livestock were kept (years)	1 = <1 2 = 1–3 3 = 4–5 4 = 6–10 5 = >10	1 (2.4) 4 (9.5) 5 (11.9) 10 (23.1) 22 (52.4)	32.5	4	<0.001 ***
Raising in the same route with other herds	0 = no 1 = yes	26 (61.9) 16 (38.1)	2.4	1	0.12 ^ns^
Sharing feeding ground with other herds	0 = no 1 = yes	16 (38.1) 26 (61.9)	2.4	1	0.12 ^ns^
Introduced of new animal into herd	0 = no 1 = yes	31 (73.8) 11 (26.2)	9.5	1	0.002 **
Pets in the farm	0 = no 1 = yes	9 (21.42) 33 (78.6)	18.7	1	<0.001 ***
Abortion history	0 = not aborted 1 = aborted	27 (64.3) 15 (35.7)	3.4	1	0.06 ^ns^

^a^ Significant level: ns = not significant, * = 0.05, ** = 0.01, *** = 0.001.

**Table 4:** Factor loading eigenvalue, environmental factors, seroprevalence, and coordinates of sites of correlative coherence analysis (CCA) around the Salakphra Wildlife Sanctuary.

Parameter	PC1	PC2	PC3
Risk factor			
Location of herd *	−0.265	0.062	−0.165
Herd size	−0.017	−0.146	−0.054
Place	−0.100	−0.031	0.098
Raising distance (km/day) *	−0.076	0.111	0.081
Together	−0.080	−0.251	0.029
Togeth_a	−0.061	0.069	0.088
Number of years that domestic cattle were kept (years) *	0.032	0.250	−0.241
Introduced of new animal into herd *	−0.257	0.052	0.015
Abortion history *	−0.094	−0.223	−0.014
Domestic cattle	0.099	0.200	0.103
*Leptospira interrogans* in domestic cattle			
Autumnalis	−2.948	−0.884	−0.673
Ballum	−0.380	−0.293	0.617
Bataviae	0.831	−0.853	0.345
Bratislava	0.758	−0.695	−2.133
Canicola	−0.770	4.907	0.283
Cynopteri	−1.054	−1.306	0.478
Djasiman	−2.056	−0.632	−0.288
Hebdomadis	0.483	−0.274	−1.459
Icterohaemorrhagica	−3.320	−1.305	−0.360
Javanica	−4.350	−0.279	−2.689
Louisiana	0.982	−3.859	7.542
Mini	−2.176	3.175	−0.534
Pomona	−1.037	1.766	−1.483
Pyrogenes	−0.994	4.179	0.566
Ranarum	1.033	0.209	−0.066
Sarmin	−1.804	1.108	−1.141
Sejroe	0.041	−0.602	2.720
Tarassovi	0.129	−0.160	−0.142

* Denotes significant relationship at *p* < 0.05.
